# Evaluation of Clinical Outcome and Survival Under Application of Various Therapies at First Recurrence in Patients with Glioblastoma

**DOI:** 10.3390/jcm14186550

**Published:** 2025-09-17

**Authors:** Marion Rapp, Hannah Fischer, Julia Steinmann, Michael Sabel, Franziska Staub-Bartelt

**Affiliations:** 1 Department of Neurosurgery, University Hospital Düsseldorf, 40225 Düsseldorf, Germany; 2 Medical Faculty, Heinrich-Heine-University Düsseldorf, 40225 Düsseldorf, Germany

**Keywords:** glioblastoma, recurrence, recurrent surgery, re-radiation, brain tumor, temozolomide

## Abstract

**Background:** Glioblastoma (GBM) patients exhibit a median overall survival of 12–18 months post-diagnosis, with disease recurrence typically emerging within 6–9 months. Due to the absence of standardized therapeutic protocols at recurrence, management is highly individualized. This study comprehensively evaluates overall survival (OS) time to subsequent progression, and clinical status evolution following diverse interventions for first GBM recurrence. **Methods:** Data from 350 patients were retrospectively analyzed. The entire cohort was divided into the following four groups: (A) patients with no further therapy at recurrence, (B) combined re-radiation and chemotherapy with temozolomide with or without lomustine or other individual medication, (C) surgery without re-adjuvant treatment, and (D) surgery and at least one cycle of chemotherapy or re-radiation or a combination. Statistical analyses were performed using non-parametric tests. Additionally, various regression analyses were performed. **Results:** Patients receiving invasive therapeutic regimens with or without adjuvant re-therapy (groups C and D) demonstrated significantly prolonged OS (*p* < 0.001) alongside superior Karnofsky performance status (KPS) at both 3-month (*p* = 0.016) and 6-month (*p* < 0.001) intervals post-intervention. Multivariate analysis confirmed surgical resection, temozolomide (TMZ) chemotherapy, and radiotherapy as independent positive predictors of OS (respective *p*-values: <0.001, <0.001, and 0.048). Notably, surgical resection significantly improved clinical status (*p* < 0.001), whereas radiotherapy had a significant negative effect on clinical status (*p* = 0.016). **Conclusions:** Contrary to the prevailing hypothesis that survival extension through extensive therapy at recurrence necessitates compromised clinical status, our findings demonstrate that contemporary recurrence therapies—particularly multimodal approaches—simultaneously enhance both OS and functional outcomes in GBM patients. This paradigm challenges conventional expectations of therapeutic trade-offs at disease recurrence.

## 1. Introduction

Despite all scientific efforts over the last decades, glioblastoma—the most common malignant brain tumor—still has a median survival of only 1 to 1.5 years after initial diagnosis [1] and a 5-year survival rate of 6.8% in the United States [2]. Median time to recurrence is reported as 6–9 months [3,4]. Tumor progression should be considered when patients with glioblastoma experience new or worsened neurological symptoms [5] or undergo regular imaging procedures, particularly MR imaging, which detect tumor growth [6,7]. After recurrence diagnosis, patients undergo an individualized therapy regimen including standard approaches, such as re-surgery, re-radiation, and/or chemotherapy with temozolomide (TMZ) or off-label therapies within studies, as there is currently no established standard therapy for recurrence [4,8]. Depending on comorbidities and previous therapies, the combination of surgical resection, radiotherapy, and chemotherapy is usually used [9]. As well as at the time of initial diagnosis, surgical resection aims to remove as much of the tumor as possible while preserving healthy tissue and preventing postoperative neurological deterioration [10,11,12]. Hereby, the potentially altered anatomical situation compared to the initial surgery might complicate the surgical approach in recurrence [13]. Adjuvant therapy with radiotherapy and chemotherapy may be less effective when reapplied in the event of recurrence, as resistance may already have developed, limiting the effectiveness of the therapy [14,15]. In addition, the increased radiation dose often leads to radionecrosis [16]. Those therapies, however, serve to remove (surgery) or control (radio-chemotherapy) as much of the tumor as possible to prolong OS and the progression-free interval. Before choosing recurrent therapy regimes, the clinical status and the impact of the various therapy options on quality of life must be taken into consideration. For the assessment of the clinical status, the Karnofsky performance index (KPS) can be used [17]. Especially in groups with a lower KPS, the score can also be used as a surrogate parameter for quality of life [18].

The present study investigated which therapy offers the best chances of positively influencing survival time in terms of OS and progression-free interval, as well as quality of life (QoL) in patients with first recurrence of GBM.

## 2. Methods

### 2.1. Study Design

**Figure 1:**
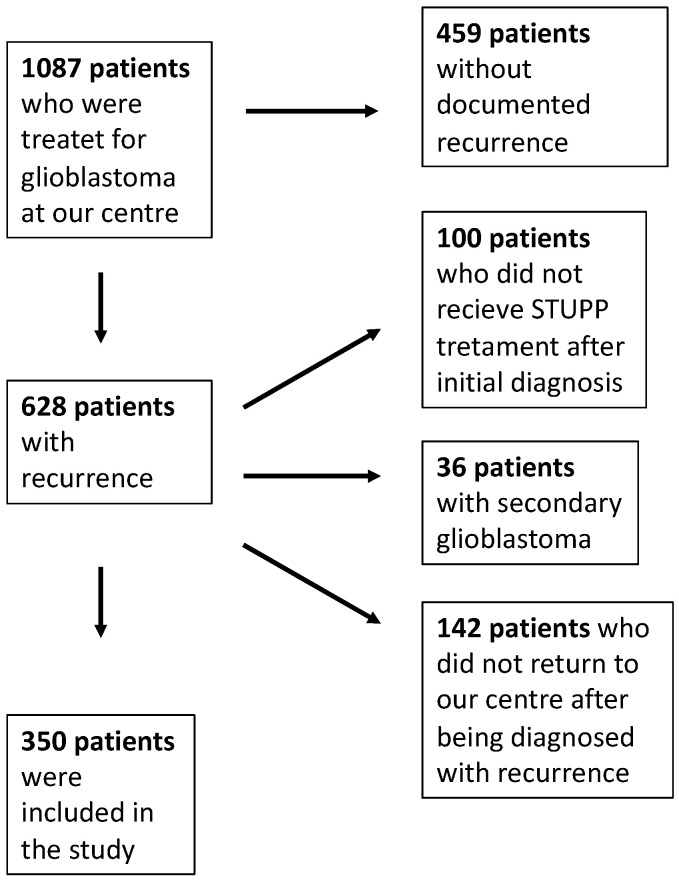
Patient selection within this study.

We conducted a retrospective, single-center cohort study including adult patients (>18 years) with histologically confirmed glioblastoma treated at the neurosurgical department of the University Hospital in Duesseldorf, Germany, between 2010 and 2021 for their first glioblastoma recurrence. The study was performed and reported in line with the STROBE statement for observational research. Patients were excluded if they (1) did not experience a documented recurrence, (2) had not received the standard-of-care Stupp protocol [19] at initial diagnosis, or (3) were diagnosed with secondary glioblastoma. In addition, patients who did not return to our center for treatment or follow-up after diagnosis of recurrence were excluded due to the absence of reliable data on subsequent management and outcomes. This step was necessary to ensure a homogeneous study population with standardized treatment and follow-up, allowing for robust and consistent outcome analysis. The entire cohort was divided into four groups depending on the therapy received for the first recurrence. Group A (n = 75, 21.4%) did not receive any further therapy in the form of surgery, radiation, or chemotherapy. Group B (n = 55, 15.7%) received adjuvant therapy only, consisting of a combination of radiation and chemotherapy either with temozolomide only or in combination with lomustine or the individual application of both therapy options. Group C (n = 56, 16%) received surgery without further adjuvant therapy. Group D (n = 164, 46.9%) received surgical resection of the tumor and at least one chemotherapy or radiation therapy or a combination of both adjuvant therapies. Figure 1 shows a flowchart of the patient selection in this study.

The outcome parameters were defined as overall survival from the diagnosis of the first recurrence, progression-free interval from the diagnosis of the first recurrence to the diagnosis of the second recurrence, and KPS. Date of MR imaging with signs of recurrence was used as the date of diagnosis for first or second recurrence, independently of the start of any clinical symptoms. The KPS was recorded at the time of diagnosis of first recurrence and follow-up after three and six months and was documented in the clinical records. While the KPS is a well-established and widely applied measure of physical performance and independence in neuro-oncology, it is a unidimensional tool. It does not comprehensively capture other relevant aspects of quality of life, such as cognitive function, psychological wellbeing, symptom burden, or social and role functioning. Therefore, results reported in connection with the KPS should be interpreted as describing functional status rather than a multidimensional assessment of quality of life. Variables for Cox regression analysis comprised sociodemographic factors, tumor volume, and survival time. Furthermore, we investigated localization of the primary tumor, eloquence of the tumor measured by intraoperatively detected functionability, confirmation of recurrence diagnosis, and localization of the recurrent tumor.

The diagnosis of recurrence was established according to RANO criteria, based on MRI findings of progressive or new enhancing lesions in combination with clinical deterioration. In cases with equivocal imaging, advanced MRI techniques (such as perfusion and spectroscopy) were applied, and histopathological confirmation was obtained when available.

Approval was obtained from the local ethics committee (Study-Nr 2023-2480) before the start of the study.

### 2.2. Statistical Analysis

Statistical analysis was performed using SPSS Version 29 (IBM SPSS Statistics (Version 29.0.2.0), IBM, Armonk, NY, USA). Descriptive statistics were calculated for all variables. Survival time data were analyzed using Kaplan–Meier curves and the log-rank test. In case of a missing death date or date of diagnosis of the second recurrence, the corresponding data were censored. For the inclusion of multiple predictor variables, we additionally performed Cox regression analysis. Data that were not normally distributed according to the Kolmogorov–Smirnov test, the non-parametric Kruskal–Wallis test, and the Mann–Whitney U test were used for group comparison. Regression analysis was also performed to evaluate the influence of different factors that might influence the data. In general, statistical significance was assumed at a *p*-value of 0.05 or lower.

## 3. Results

A total of 350 patients met the inclusion criteria, of whom 60% were male patients (male n = 218, female n = 132). Mean age at time of first recurrence was 59.83 years (SD ± 12.63). Median time to first recurrence was 7 months (IQR 4–14 months). A total of 132 patients showed second recurrence within a median time of 6 months after first recurrence (IQR 2–7 months). Median time of survival from first recurrence was 7 months (IQR 3–12 months). The median KPS in the general cohort was 90%.

Of all patients included in the study, 21% (n = 75) belonged to group A, 16% (n = 55) belonged to group B, 16% (n = 56) belonged to group C, and 47% (n = 164) belonged to group D.

Table 1 presents the sociodemographic characteristics of the four study groups (A–D). Significant differences were observed between the groups regarding the median age at the time of recurrence (group A: 67 years; B: 59 years; C: 59 years; D: 60 years; *p* < 0.001) as well as the median residual tumor volume after surgical treatment of the recurrent lesion (group C: 2.31 cm^3^; group D: 0.77 cm^3^; *p* = 0.016).

The clinical condition, assessed using the Karnofsky performance status (KPS), also differed significantly across all three time points, namely time of recurrent diagnosis: A = 80, B = 90, C = 90, and D = 90; *p* < 0.001; at the 3-months follow-up: A = 70, B = 80, C = 90, and D = 90; *p* < 0.001; and at the six-months follow-up: A = 0, B = 0, C = 40, and D = 70; *p* < 0.001.

**Figure 2:**
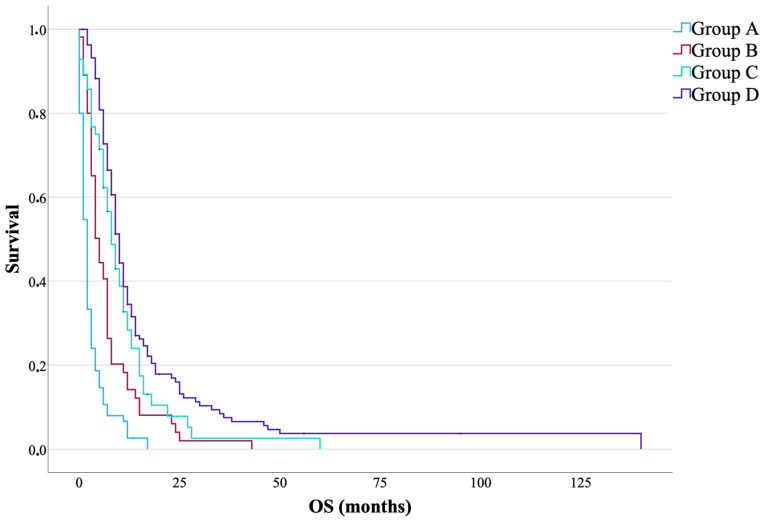
Kaplan–Meier overall survival curves for patients with first glioblastoma recurrence, stratified by treatment group. Group A: no further therapy (n = 75); group B: adjuvant therapy only (chemotherapy and/or radiotherapy, n = 55); group C: surgery without adjuvant therapy (n = 56); group D: surgery combined with adjuvant therapy (n = 164). Patients censored at the last follow-up are indicated by crosses. Median overall survival differed significantly between the groups (group A: 2 months, group B: 5 months, group C: 8 months, and group D: 10 months; log-rank *p* < 0.001).

The median overall survival (OS), measured in months from the time of first recurrence, showed significant differences between the analyzed groups, as follows: group (2 months), group B (5 months), group C (8 months), and group D (10 months) (*p* < 0.001). Figure 2 shows a Kaplan–Meier plot illustrating OS across different treatment groups.

Additionally, tumor localization and recurrence characteristics were investigated. At first recurrence, tumor localization was most frequently observed in the frontal lobe (n = 125) and parietal lobe (n = 103). In total, 303 patients presented with focal tumor growth at recurrence, while data were missing in 13 cases. Among the patients who underwent resection, the tumor was located in an eloquent area in 145 cases. Confirmation of recurrence was achieved histologically in 220 patients, by FET-PET in 32 patients, and by RANO criteria alone in the remaining cases. In patients who developed a second recurrence (n = 165), the tumor again occurred at a focal location.

At recurrence, different chemotherapy regimens were applied, including the standard concomitant temozolomide protocol (75 mg/m^2^; n = 110), the Perry protocol (n = 37), the Herrlinger regimen (n = 10), the one-week-on/one-week-off schedule (n = 7), and other protocols (n = 1). Comparative survival analyses did not reveal significant differences between protocols (OS log-rank *p* = 0.551; PFS log-rank *p* = 0.207). A significant difference was observed for functional status at recurrence diagnosis (KPS *p* = 0.033), whereas KPS at 3 and 6 months did not differ significantly (*p* = 0.59 and *p* = 0.94, respectively). Radiotherapy regimens at recurrence included 60 Gy in 2 Gy fractions (n = 12), 35 Gy in 3.5 Gy fractions (n = 3), 40 Gy in 2.67 Gy fractions (n = 56), and 31.5 Gy in 2.1 Gy fractions (n = 3). No significant differences were found between these regimens about OS (*p* = 0.12), PFS (*p* = 0.645), or KPS at diagnosis (*p* = 0.153), 3 months (*p* = 0.345), or 6 months (*p* = 0.755).

With respect to the initial surgical treatment at primary diagnosis, the mean preoperative tumor volume was 33.09 cm^3^, and the mean postoperative residual volume was 0.08 cm^3^. No significant differences were observed between treatment groups regarding the extent of initial resection.

Further analysis revealed that patients undergoing surgery showed significantly better survival (median OS) and significantly better clinical status (KPS) at all three assessed timepoints (median OS *p* < 0.001; KPS *p* < 0.001). Additionally, chemotherapy with TMZ and radiotherapy was associated with a statistically significant improvement in median OS in all cases (TMZ *p* < 0.001, radiotherapy *p* = 0.048). Chemotherapy demonstrated a positive impact on functional status, as reflected by a significantly higher KPS measured six months after the initiation of treatment for the first recurrence (*p* < 0.001). In contrast, radiotherapy was associated with a significantly lower median KPS by approximately 10 points three months after treatment initiation compared to the group that did not receive radiotherapy (*p* = 0.016). The results are summarized in Table 2.

Further regression analysis of the statistical impact of MGMT-status, age and clinical status at time of recurrent diagnosis, as well as residual tumour volume on OS, PFS and KPS was performed. Here, MGMT status showed a significant influence on PFS (*p* = 0.022). Both the patient’s age at the time of first recurrence diagnosis and the residual tumour volume following surgical resection of the first recurrence were significant predictors of OS (age: *p* < 0.001, residual tumour volume: *p* < 0.001). Concerning PFS, the residual tumour volume still had a significant impact (*p* = 0.005). Furthermore, both variables showed a statistically significant association with the KPS six months after treatment in simple regression analysis (age: *p* < 0.001, residual tumour volume: *p* = 0.049). For detailed results, refer to the following Table 3 (Cox regression) and Table 4 (simple/multiple regression).

## 4. Discussion

To identify the most effective standard treatment strategies for patients with a first recurrence of glioblastoma, we conducted a retrospective, single-center analysis of 350 patients. The demographic and clinical characteristics of our cohort—particularly in terms of age, sex distribution, and functional status—are largely consistent with those reported in comparable studies [20,21,22]. A key distinction of our study is that, in contrast to many previous investigations, such as the study by Clavreul et al. [21], which focused on the effect of a single treatment modality, we provide both a combined analysis of various treatment strategies and an assessment of the individual effects of each therapeutic option. Additionally, our study includes a comparison group that did not undergo further treatment. In many comparable studies, treatment arms typically involve combinations of active interventions, thereby limiting the ability to evaluate the isolated effect of treatment versus no treatment. A statistically significant difference in OS was observed between groups A and D, with a clear trend indicating that increased therapeutic intensity is associated with prolonged survival following diagnosis of recurrence. Despite methodological differences, the findings of De Bonis et al. similarly suggest that more extensive treatment is correlated with improved OS [23]. Vice versa, van Linde et al. [24] analyzed 299 patients and included a best supportive care group, reporting substantially shorter survival in these patients compared to those undergoing re-resection or active therapies. Similarly, Archavlis et al. [22] demonstrated superior survival after local invasive approaches, with median overall survival of 37 weeks after HDR brachytherapy and 30 weeks after reoperation, compared to 26 weeks with temozolomide alone. These results are consistent with our observation that surgical resection was strongly associated with prolonged survival and improved functional status.

Among the treatment modalities evaluated, surgical resection demonstrated the most pronounced impact on both OS and clinical status (KPS) when compared to the group that did not undergo surgery. This finding is consistent with the results reported by Voisin et al. [25]. However, the isolated effect of chemotherapy is more challenging to interpret, as the majority of patients in the first recurrence setting received temozolomide-based regimens. A relevant comparison can be drawn from a study in which OS was analyzed between a group receiving combined chemoradiotherapy and a group treated with radiotherapy alone. This study likewise highlights the beneficial effect of temozolomide on OS [26]. The impact of radiotherapy on patient outcomes was also analyzed. While radiotherapy was associated with a significant improvement in OS, the clinical condition, measured by KPS, was significantly better in the group that did not receive radiotherapy. Findings on radiotherapy and OS are consistent with results from other studies. For instance, the publication by Marwah et al. [27] not only confirmed the positive effect of radiotherapy on OS but also demonstrated a beneficial influence on the PFS. A Cochrane network meta-analysis [15] evaluated a wide range of interventions and reported median overall survival between 5.5 and 12.6 months across studies, most frequently with systemic treatments, such as lomustine. However, surgical and radiotherapeutic interventions were underrepresented, limiting the strength of conclusions for these modalities. Our data contribute by highlighting the significant impact of surgery and multimodal strategies in a large single-center cohort.

An apparent paradox observed in our cohort was that re-irradiation was associated with improved OS while at the same time leading to a decline in short-term functional status as measured by KPS. The observed change in KPS is supported by findings from another study, which also reported a decline in median KPS following radiotherapy. Although the exact numerical change was not specified, the overall trend aligns with our results. However, a notable limitation of our analysis is that a decline in median KPS was also observed in the group that did not receive radiotherapy. Importantly, the referenced study identified a correlation between the irradiated volume and the extent of clinical deterioration, suggesting that treatment-associated morbidity may be dose-dependent [28]. In addition, radiation-induced changes, such as fatigue, radionecrosis, or transient oedema, can compromise performance status in the short term, even though tumor control achieved by re-irradiation may prolong survival. These findings highlight the importance of carefully balancing potential survival benefits against the risk of functional deterioration when selecting patients for re-irradiation

Additionally, our study demonstrated that the clinical condition remains stable despite the use of invasive therapeutic interventions. This observation is supported by the findings of Ringel et al. [20], who assessed KPS immediately postoperatively. Although the timing of the assessment differed from our study, their results likewise indicate a stable postoperative functional status.

Finally, we want to mention the other important aspects when discussing recurrence therapy and survival. Firstly, a well-known relevant prognostic factor in glioblastoma is the extent of the initial resection at first diagnosis, which is well established as an independent predictor of overall survival [29]. In our cohort, mean preoperative and postoperative tumor volumes at initial surgery were recorded, demonstrating a very low mean residual volume (0.08 cm^3^) and no significant differences between the treatment groups. Nevertheless, we did not categorize the initial procedures explicitly into gross total versus subtotal resections. We acknowledge this as a limitation, as the absence of a more detailed classification may reduce the comparability with studies that stratified patients according to resection type. However, the uniformly low residual tumor volumes observed suggest that most patients underwent extensive resections, which may have mitigated the potential impact of this variable on the outcomes reported.

Secondly, in addition to the standard re-intervention modalities analyzed in our study (surgery, chemotherapy, and radiotherapy), several other therapeutic avenues are currently under active investigation and merit mention. Angiogenesis inhibitors, most notably bevacizumab, have been studied extensively in first-diagnosis and recurrent glioblastoma, though findings suggest improved progression-free survival at best, with overall survival benefits remaining equivocal [30,31]. Immunotherapy is similarly an evolving frontier, and strategies, such as immune checkpoint inhibitors, adoptive T-cell therapies, cancer vaccines, and oncolytic viral therapies, demonstrate promising early-phase results, albeit while facing significant challenges in immune resistance and delivery [32]. Moreover, innovative approaches, including stereotactic radiosurgery combined with targeted agents (e.g., radiosurgery with bevacizumab), are under exploration and may offer improved focal control with limited toxicity [4,33,34] These emerging modalities, while not the focus of our retrospective analysis, underscore the dynamic therapeutic landscape and should be considered in future prospective studies aiming to improve outcomes and functional preservation in recurrent GBM.

## 5. Limitations

As a retrospective study, our analysis was based exclusively on data already available at the time of study initiation. Consequently, no potentially relevant or necessary information could be collected retrospectively. This limitation is particularly relevant in the context of the patient population and observation period, as the majority of patients were already deceased by the time data were extracted from clinical information systems. In addition to this, due to the study’s retrospective character, we conducted a non-randomized study, which entails a risk of selection bias, particularly with respect to group A, where patients did not receive further active therapy at recurrence. This subgroup predominantly consisted of individuals with poor performance status or relevant comorbidities, which inherently predisposed them to worse outcomes. Therefore, while our findings support the potential benefit of aggressive interventions in appropriately selected patients, they should not be interpreted as a direct comparison of therapeutic efficacy across all groups but rather as real-world evidence within clinically distinct subpopulations. Notably, retrospective collection of clinical information, such as quality-of-life data or patient-reported outcomes, is not feasible. Moreover, although KPS provides an estimate of the patient’s functional status, it does not fully capture the multidimensional concept of quality of life, which also includes psychological, social, and subjective factors [35].

An additional methodological limitation arises from the non-randomized study design. Patients were allocated to specific treatment modalities based on individual characteristics, such as age, clinical performance, and location of recurrence. As a result, selection bias cannot be excluded. It is likely that patients in group A, who did not receive further active treatment in the form of surgical resection, chemotherapy, or radiotherapy, already had a worse prognosis due to ineligibility for invasive therapies. This inherent imbalance in baseline characteristics may have influenced the observed outcome differences between treatment groups. Furthermore, the exclusion of a substantial proportion of patients from the initial cohort, particularly the subgroup of patients (n = 142) who did not return to our center after being diagnosed with recurrence, limits the generalizability of our findings to the broader glioblastoma population, as their absence may introduce selection bias. However, the restriction to patients treated and followed longitudinally at a single tertiary referral center ensured consistent diagnostic work-up, treatment decisions, and follow-up protocols, thereby strengthening the internal validity of our analysis. A further limitation arises from the lack of uniformly applied standardized criteria to distinguish true recurrence from pseudoprogression. Although we relied on RANO criteria, advanced imaging, and histopathological confirmation when feasible, the retrospective design may still have introduced a risk of misclassification.

## 6. Conclusions

In summary, our study provides evidence that invasive therapeutic approaches can be beneficial to patients with recurrent glioblastoma who are in good clinical condition. Contrary to concerns that extensive interventions, such as maximal safe resection with minimal residual tumor volume or multimodal adjuvant therapy, might lead to significant clinical deterioration, our findings suggest that these strategies are associated with a preservation of functional status.

## Figures and Tables

**Table 1:** Descriptive sociodemographic characteristics of the study cohort, as well as clinical data, such as MGMT-Status, residual tumor volume, and outcome parameters, like OS, PFS, and KPS for all 4 groups, “*” indicates significant results (*p* < 0.05).

Characteristic	Group A	Group B	Group C	Group D	*p*-Value
Number	75 (21.4%)	55 (15.7%)	56 (16%)	164 (46.9%)	
Sex					
Female	33 (44%)	19 (35%)	26 (46%)	54 (33%)	
Male	42 (56%)	36 (65%)	30 (54%)	110 (67%)	
Age at diagnosis of recurrence in years (median)	67	59	59	60	<0.001 *
MGMT-Status					
Positive	26 (35%)	11 (20%)	16 (29%)	45 (27%)	
Negative	33 (44%)	30 (55%)	30 (54%)	79 (48%)	
Tumor volume of recurrence in cm^3^ (mean)	15.65	12.84	12.72	11.17	0.163
Residual tumor volume after surgery in cm^3^ (medium)			2.31	0.77	0.016 *
Overall survival in months (median)	2	5	8	10	<0.001 *
Progression-free interval in months (median)			5	6	0.069
KPS in case of recurrence diagnosis	80	90	90	90	<0.001 *
KPS after 3 months	70	80	90	90	<0.001 *
KPS after 6 months	0	0	40	70	<0.001 *

**Table 2:** Statistical impact of different therapy modalities on survival (OS and PFS) as well as clinical status (KPS) of the patients. Surgery was part of the therapy in groups C and D. Chemotherapy and radiotherapy were part of the therapy in groups B and D, “*” indicates significant results (*p* < 0.05).

	Surgery			Temozolomide			Radiotherapy		
	Yes	No	*p*-Value	Yes	No	*p*-Value	Yes	No	*p*-Value
OS (median)	9	3	<0.001 *	9	4	<0.001 *	9	6	0.048 *
PFS (median)	6	9	0.007 *	6	7	0.962	7	6	0.808
KPS PD (median)	90	80	<0.001 *	90	90	0.007 *	90	90	<0.001 *
KPS 3 M (median)	90	80	<0.001 *	90	80	0.354	70	80	0.016 *
KPS 6 M (median)	70	0	<0.001 *	70	0	<0.001 *	0	0	<0.001 *

**Table 3:** Cox regression analysis results showing significant influence of age and residual tumor volume on OS (age *p* < 0.001, residual tumor volume *p* < 0.001). Residual tumor volume also had a significant impact on PFS (*p* = 0.005), as well as MGMT status (*p* = 0.022), “*” indicates significant results (*p* < 0.05).

Influence Factor	Dependent Variable	*p*-Wert Cox Regression
MGMT	OS	0.197
	PFS	0.022 *
Age	OS	<0.001 *
	PFS	0.541
Clinical status	OS	0.749
	PFS	0.484
Residual tumor volume	OS	<0.001 *
	PFS	0.005 *

**Table 4:** Simple and multiple regression analysis to evaluate the impact of MGMT status, age, clinical status at recurrent diagnosis, and residual tumor volume post-recurrent resection on KPS. Age significantly impacted KPS at six months in the simple regression analysis (*p* < 0.001). The initial clinical status at recurrent diagnosis furthermore significantly influenced KPS at three (*p* < 0.001) and six months follow-up (*p* < 0.001) in the simple regression analysis. KPS at six months was also significantly influenced by residual tumor volume in the simple (*p* = 0.049) and multiple regression analyses (*p* = 0.046), “*” indicates significant results (*p* < 0.05).

Influence Factor	Dependent Variable	Test	*p*-Value
MGMT	KPS after three months	Simple linear regression	0.504
		Multiple linear regression	0.611
	KPS after six months	Simple linear regression	0.632
		Multiple linear regression	0.847
Age	KPS after three months	Simple linear regression	0.067
		Multiple linear regression	0.574
	KPS after six months	Simple linear regression	<0.001 *
		Multiple linear regression	0.059
Clinical status (at the time of the diagnosis of first recurrence)	KPS after three months	Simple linear regression	<0.001 *
		Multiple linear regression	0.009 *
	KPS after six months	Simple linear regression	<0.001 *
		Multiple linear regression	0.927
Residual tumor volume	KPS after three months	Simple linear regression	0.706
		Multiple linear regression	0.768
	KPS after six months	Simple linear regression	0.049 *
		Multiple linear regression	0.046 *

## Data Availability

Data available on request due to restrictions (e.g., privacy, legal or ethical reasons). The data presented in this study are available on request from the corresponding author due to data share restrictions (ethic committee).

## References

[1] Krex D., Schackert G. (2017). Glioblastome. Gliomchirurgie.

[2] Ostrom Q.T., Patil N., Cioffi G., Waite K., Kruchko C., Barnholtz-Sloan J.S. (2020). CBTRUS Statistical Report: Primary Brain and Other Central Nervous System Tumours Diagnosed in the United States in 2013–2017. Neuro Oncol..

[3] Birzu C., French P., Caccese M., Cerretti G., Idbaih A., Zagonel V., Lombardi G. (2020). Recurrent Glioblastoma: From Molecular Landscape to New Treatment Perspectives. Cancers.

[4] Vaz-Salgado M.A., Villamayor M., Albarrán V., Alía V., Sotoca P., Chamorro J., Rosero D., Barrill A.M., Martín M., Fernandez E. (2023). Recurrent Glioblastoma: A Review of the Treatment Options. Cancers.

[5] Alentorn A., Hoang-Xuan K., Mikkelsen T. (2016). Presenting signs and symptoms in brain tumours. Handb. Clin. Neurol..

[6] Himes B.T., Arnett A.L., Merrell K.W., Gates M.J., Bhargav A.G., Raghunathan A., Brown D.A., Burns T.C., Parney I.F. (2020). Glioblastoma Recurrence Versus Treatment Effect in a Pathology-Documented Series. Can. J. Neurol. Sci..

[7] Youssef G., Rahman R., Bay C., Wang W., Lim-Fat M.J., Arnaout O., Bi W.L., Cagney D.N., Chang Y.S., Cloughesy T.F. (2023). Evaluation of Standard Response Assessment in Neuro-Oncology, Modified Response Assessment in Neuro-Oncology, and Immunotherapy Response Assessment in Neuro-Oncology in Newly Diagnosed and Recurrent Glioblastoma. J. Clin. Oncol..

[8] Sacko O., Benouaich-Amiel A., Brandicourt P., Niaré M., Charni S., Cavandoli C., Brauge D., Catalaa I., Brenner A., Moyal E.C. (2021). The Impact of Surgery on the Survival of Patients with Recurrent Glioblastoma. Asian J. Neurosurg..

[9] Alirezaei Z., Amouheidari A., BasirianJahromi R., Seyyedhosseini S., Hamidi A. (2025). Survival Analysis of Glioblastoma: A Scientometric Perspective. World Neurosurg..

[10] Gerritsen J.K.W., Broekman M.L.D., De Vleeschouwer S., Schucht P., Nahed B.V., Berger M.S., Vincent A. (2022). Safe surgery for glioblastoma: Recent advances and modern challenges. Neurooncol. Pract..

[11] Rahman M., Abbatematteo J., De Leo E.K., Kubilis P.S., Vaziri S., Bova F., Sayour E., Mitchell D., Quinones-Hinojosa A. (2017). The effects of new or worsened postoperative neurological deficits on survival of patients with glioblastoma. J. Neurosurg..

[12] McGirt M.J., Mukherjee D., Chaichana K.L., Than K.D., Weingart J.D., Quinones-Hinojosa A. (2009). Association of surgically acquired motor and language deficits on overall survival after resection of glioblastoma multiforme. Neurosurgery.

[13] Yang K., Ellenbogen Y., Martyniuk A., Sourour M., Takroni R., Somji M., Gardiner E., Hui K., Odedra D., Larrazabal R. (2022). Reoperation in adult patients with recurrent glioblastoma: A matched cohort analysis. Neurooncol. Adv..

[14] Chang C., Chavarro V.S., Gerstl J.V.E., Blitz S.E., Spanehl L., Dubinski D., Valdes P.A., Tran L.N., Gupta S., Esposito L. (2024). Recurrent Glioblastoma-Molecular Underpinnings and Evolving Treatment Paradigms. Int. J. Mol. Sci..

[15] McBain C., Lawrie T.A., Rogozińska E., Kernohan A., Robinson T., Jefferies S. (2021). Treatment options for progression or recurrence of glioblastoma: A network meta-analysis. Cochrane Database Syst. Rev..

[16] Fernandes C., Costa A., Osório L., Lago R.C., Linhares P., Carvalho B., Caeiro C., De Vleeschouwer S. (2017). Current Standards of Care in Glioblastoma Therapy. Glioblastoma.

[17] Terret C., Albrand G., Moncenix G., Droz J.P. (2011). Karnofsky Performance Scale (KPS) or Physical Performance Test (PPT)? That is the question. Crit. Rev. Oncol. Hematol..

[18] Mackworth N., Fobair P., Prados M.D. (1992). Quality of life self-reports from 200 brain tumour patients: Comparisons with Karnofsky performance scores. J. Neurooncol..

[19] Stupp R., Mason W.P., van den Bent M.J., Weller M., Fisher B., Taphoorn M.J., Belanger K., Brandes A.A., Marosi C., Bogdahn U. (2005). Radiotherapy plus concomitant and adjuvant temozolomide for glioblastoma. N. Engl. J. Med..

[20] Ringel F., Pape H., Sabel M., Krex D., Bock H.C., Misch M., Weyerbrock A., Westermaier T., Senft C., Schucht P. (2016). Clinical benefit from resection of recurrent glioblastomas: Results of a multicenter study including 503 patients with recurrent glioblastomas undergoing surgical resection. Neuro Oncol..

[21] Clavreul A., Aubin G., Delion M., Lemée J.M., Ter Minassian A., Menei P. (2021). What effects does awake craniotomy have on functional and survival outcomes for glioblastoma patients?. J. Neurooncol..

[22] Archavlis E., Tselis N., Birn G., Ulrich P., Baltas D., Zamboglou N. (2013). Survival analysis of HDR brachytherapy versus reoperation versus temozolomide alone: A retrospective cohort analysis of recurrent glioblastoma multiforme. BMJ Open.

[23] De Bonis P., Fiorentino A., Anile C., Balducci M., Pompucci A., Chiesa S., Sica G., Lama G., Maira G., Mangiola A. (2013). The impact of repeated surgery and adjuvant therapy on survival for patients with recurrent glioblastoma. Clin. Neurol. Neurosurg..

[24] van Linde M.E., Brahm C.G., de Witt Hamer P.C., Reijneveld J.C., Bruynzeel A.M.E., Vandertop W.P., van de Ven P.M., Wagemakers M., van der Weide H.L., Enting R.H. (2017). Treatment outcome of patients with recurrent glioblastoma multiforme: A retrospective multicenter analysis. J. Neuro-Oncol..

[25] Voisin M.R., Zuccato J.A., Wang J.Z., Zadeh G. (2022). Surgery for Recurrent Glioblastoma Multiforme: A Retrospective Case Control Study. World Neurosurg..

[26] Athanassiou H., Synodinou M., Maragoudakis E., Paraskevaidis M., Verigos C., Misailidou D., Antonadou D., Saris G., Beroukas K., Karageorgis P. (2005). Randomized phase II study of temozolomide and radiotherapy compared with radiotherapy alone in newly diagnosed glioblastoma multiforme. J. Clin. Oncol..

[27] Marwah R., Xing D., Squire T., Soon Y.Y., Gan H.K., Ng S.P. (2023). Reirradiation versus systemic therapy versus combination therapy for recurrent high-grade glioma: A systematic review and meta-analysis of survival and toxicity. J. Neuro-Oncol..

[28] Demogeot N., Salleron J., Rech F., Taillandier L., Royer P., Vogin G. (2022). Impact of fractionated stereotactic radiotherapy on activity of daily living and performance status in progressive/recurrent glioblastoma: A retrospective study. Radiat. Oncol..

[29] Karschnia P., Young J.S., Dono A., Häni L., Sciortino T., Bruno F., Juenger S.T., Teske N., Morshed R.A., Haddad A.F. (2023). Prognostic validation of a new classification system for extent of resection in glioblastoma: A report of the RANO resect group. Neuro Oncol..

[30] Zhang T., Xin Q., Kang J.M. (2021). Bevacizumab for recurrent glioblastoma: A systematic review and meta-analysis. Eur. Rev. Med. Pharmacol. Sci..

[31] Gilbert M.R., Dignam J.J., Armstrong T.S., Wefel J.S., Blumenthal D.T., Vogelbaum M.A., Colman H., Chakravarti A., Pugh S., Won M. (2014). A randomised trial of bevacizumab for newly diagnosed glioblastoma. N. Engl. J. Med..

[32] Liu Y., Zhou F., Ali H., Lathia J.D., Chen P. (2024). Immunotherapy for glioblastoma: Current state, challenges, and future perspectives. Cell. Mol. Immunol..

[33] Hundsberger T., Reardon D.A., Wen P.Y. (2017). Angiogenesis inhibitors in tackling recurrent glioblastoma. Expert. Rev. Anticancer Ther..

[34] Rizwani F., Patil P., Jain K. (2025). Unlocking glioblastoma: Breakthroughs in molecular mechanisms and next-generation therapies. Med. Oncol..

[35] Schaafsma J., Osoba D. (1994). The Karnofsky Performance Status Scale re-examined: A cross-validation with the EORTC-C30. Qual. Life Res..

